# Adalimumab biosimilars in the therapy of Crohn´s disease and ulcerative colitis: Prospective multicentric clinical monitoring

**DOI:** 10.1371/journal.pone.0271299

**Published:** 2022-08-08

**Authors:** Martin Wasserbauer, Stepan Hlava, Jiri Drabek, Jan Stovicek, Petra Minarikova, Lenka Nedbalova, Tomas Drasar, Zdena Zadorova, Jiri Dolina, Stefan Konecny, Vladimír Kojecky, Jana Kozeluhova, Pavlina Cernikova, Dita Pichlerova, Barbora Kucerova, Stepan Coufal, Radan Keil

**Affiliations:** 1 Department of Internal Medicine, 2nd Faculty of Medicine, Motol University Hospital, Charles University in Prague, Prague, Czech Republic; 2 Department of Internal Medicine, 1st Faculty of Medicine, Military University Hospital, Charles University in Prague, Prague, Czech Republic; 3 Department for the Treatment of Non-specific Intestinal Inflammations - IBD Center Turnov, Hospital Turnov, Turnov, Czech Republic; 4 2nd Department of Internal Medicine, 3rd Faculty of Medicine, FNKV, Charles University in Prague, Prague, Czech Republic; 5 Department of Internal Medicine and Gastroenterology, Faculty of Medicine, University Hospital Brno, Masaryk University, Brno, Czech Republic; 6 Department of Internal Medicine, Regional Hospital of T. Baťa, Zlín, Czech Republic; 7 2nd Department of Internal Medicine, University Hospital Plzeň - Bory, Plzeň, Czech Republic; 8 Department of Pediatric Surgery, 2nd Faculty of Medicine, University Hospital Motol, Charles University in Prague, Prague, Czech Republic; 9 Laboratory of Cellular and Molecular Immunology, Institute of Microbiology of the Czech Academy of Sciences, Prague, Czech Republic; Changhua Christian Healthcare System: Changhua Christian Hospital, TAIWAN

## Abstract

**Objective:**

The adalimumab biosimilars FKB327 and GP2017 were approved for the therapy of patients with inflammatory bowel disease (IBD). Relatively few prospective studies with biosimilar adalimumab in patients with IBD have been published. The aim of this prospective observational study was to evaluate the effectiveness and safety of the biosimilar adalimumab.

**Material and methods:**

Adalimumab biosimilars FKB327 (Hulio^®^) and GP2017 (Hyrimoz^®^) were indicated to 50 naive patients in terms of biological therapy with Crohn’s disease (CD) or ulcerative colitis (UC). Effectiveness of therapy was evaluated via the Crohn’s Disease Activity Index [CDAI] or the Mayo Scoring System [MSS] in patients with CD or UC, respectively, before and after 12 weeks. Additional goals were to evaluate weight changes, laboratory tests and complications or adverse events of this therapy.

**Results:**

In CD patients, remission (CDAI <150) was achieved in 73.5% of cases, partial response (≥70-point decrease in CDAI score from baseline) in 11.8%, no response in 11.8% and 2.9% patients discontinued therapy. In UC patients, remission (total score on partial Mayo index ≤2 points) was achieved only in 18.8% of cases, partial response (≥2-point decrease in partial Mayo score from baseline) in 43.8%, no response in 25.0% and 12.5% patients discontinued therapy. There were statistically significant improvements in CDAI, MSS, haemoglobin, fecal calprotectin, albumin and CRP serum levels after 12 weeks of therapy. Seven adverse events were identified, three of which resulted in therapy being discontinued.

**Conclusions:**

This prospective observational study proved the effectiveness of the adalimumab biosimilars FKB327 and GP2017 in IBD.

## Introduction

Crohn’s disease and ulcerative colitis, which are two major forms of inflammatory bowel disease (IBD), are chronic immune-mediated diseases that are characterized by periods of remission and relapse [1, 2]. Management of IBD therapy is focused on inducing and then maintaining remission. Anti-TNFα agents, as the first used group of biological therapy, opened up a new era in IBD therapy [2]. Adalimumab, as one of the basic representatives of the group of anti-TNFα agents, is a recombinant fully humanised monoclonal IgG1 antibody directed against TNFα administered subcutaneously. The efficacy of adalimumab, repeatedly demonstrated in many studies, has found an indisputable place in the therapy of patients with especially severe forms Crohn’s disease and ulcerative colitis [3–8].

Biological drugs have a significantly more complex structure than conventional drugs. The crucial distinction is not only the primary structure of the molecule but also the secondary and tertiary structure, as well as post-translational changes, biological and immunochemical properties. Due to the complex structure of the molecule and its properties, it is practically impossible to make an identical copy of the original molecule of a biological drug. Biosimilars are not the same as generic versions of small-molecule drugs. Generics have relatively simple chemical structures and can thus be manufactured to be identical to their originator drug, in contrast to biological drugs. Biosimilar drugs can therefore be defined as highly similar drugs (not the same) to their originator biological drug [9, 10]. Determining the "high similarity" of biosimilar drugs is part of comprehensive and extensive comparability programs [9–12]. Extensive analytical tests are initially performed to confirm the high functional, physico-chemical and structural similarity of the biosimilar drug to the original product [13]. This is followed by a clinical trial phase in which comparable efficacy and safety are verified. This clinical trial is performed for a single indication of the original biological drug. When proving the effectiveness of safety in this one indication, the data are extended (so-called extrapolated) to other indications of the original biological product [14, 15].

The adalimumab biosimilars FKB327 (Hulio^®^) and GP2017 (Hyrimoz^®^) are now available for clinical use in IBD (CD and UC) in many countries all over the world. These adalimumab biosimilars have been approved in many countries in all indications as an original biological drug. Extensive laboratory non-clinical comparability exercises with FKB327 and GP2017 were made which demonstrated high similarity with original adalimumab. Subsequently, the effectiveness and safety of FKB327 and GP2017 were proved in two large clinical studies (GP2017 in patients with psoriasis and FKB327 in patients with rheumatoid arthritis) and therapeutic indications were then extrapolated to all indications as with original adalimumab [16, 17]. To this date, there are relatively few published reports regarding clinical outcomes achieved with biosimilar adalimumab in patients with CD and/or UC.

The primary outcome of this prospective observational study was to evaluate the effectiveness of FKB327 (Hulio^®^) and GP2017 (Hyrimoz^®^) in terms of response to induction treatment in patients with CD or UC after 12 weeks of therapy. The effectiveness of therapy was evaluated individually using the Crohn’s Disease Activity Index (CDAI) in patients with CD or the Mayo Scoring System (MSS) in patients with UC. The additional outcomes of this study were to evaluate the trends in values of some laboratory tests (CRP, fecal calprotectin, leukocytes, albumin and others), the weight profile of patients and endoscopic findings during therapy, complications or adverse effects of this biological therapy as well as differences in effectiveness between FKB327 (Hulio^®^) and GP2017 (Hyrimoz^®^).

## Materials and methods

### Patients

Our prospective observational study (clinical monitoring) was performed at 7 Gastroenterology departments across the Czech Republic in outpatients older than 18 years of age. The study was performed between 6/2019–8/2021.

Inclusion criteria for this study: Indication of Biosimilar adalimumab FKB327 (Hulio^®^) or GP2017 (Hyrimoz^®^) in naive patients (in terms of biological therapy) with CD or UC if indication criteria for these biological agents (according to the guidelines of the Czech Gastroenterological Society valid at the time of study) were met [18–20]. FKB327 and GP2017 were indicated in patients for treatment of:

1. CD, in case of: severe form of CD after failure of conventional therapy (especially thiopurines or methotrexate), perianal fistulas, corticodependent course of CD, high risk of unfavourable course of CD, some extraintestinal manifestations of CD and early recurrence of CD after surgical resection.
2. UC, in case of: moderate to severe active form of UC after failure of conventional therapy (especially thiopurines or methotrexate), acute severe UC attack (so-called rescue therapy), corticodependent course of UC, and some extraintestinal manifestations of UC.

Exclusion criteria for this study were: previous biological therapy, complications of IBD leading to surgery (strictures, abscess or perforations), contraindication(s) for biological therapy with adalimumab, or patient disagreement with biological therapy.

The study included patients from all participating centers who met the inclusion criteria from June 2019 to August 2021. The choice between particular type of biosimilar adalimumab (FKB327 or GP2017) depended only on availability within the individual centers involved in our study. Only one type of biosimilar adalimumab was available at each participating center (FKB327 or GP2017) and, consequently, only one biosimilar adalimumab was administered in that center. No active selection or randomization was made. Instead, the participating centers were initially chosen with respect to the type of biosimilar adalimumab administered so that the ratio between the two types of biosimilar adalimumab (as well as between the number of patients in a center) could be expected to be approximately the same and thus compared. During the study period, all patients received the treatment originally assigned at the beginning; there were no changes in the type of biosimilar adalimumab administered (FKB327 or GP2017). The minimum size of the examined group of patients was predetermined before the initialization of our study based on the recommendation of a statistician (groups of patients ≥40 due to statistical evidence) while the definitive size of our patient groups was determined by the recruitment of patients in the centers and the time schedule of the study. Subsequently, all data were collected at weeks 0 and 12 and then summarized and analysed.

Patients were allowed to use other therapy for IBD before biological therapy and concomitant therapy during therapy of biosimilar adalimumab.

### Methods

Patients fulfilling the inclusion criteria (men and women) with IBD (CD or UC) were assigned to biological therapy with FKB327 (Hulio^®^) or GP2017 (Hyrimoz^®^) in a classical induction scheme (160 mg at Week 0, 80 mg at Week 2 and then 40 mg every two weeks via subcutaneous injection). Patients were monitored for 12 weeks (7 doses of biosimilar adalimumab in total) after which they were given the option to continue with the biosimilar adalimumab therapy. However, data on treatment beyond Week 12 are not presented here.

At the beginning of clinical monitoring, patients were asked to answer some basic questions focusing on details of their disease and therapy in order to collect information on baseline demographics and characteristics. Additionally, laboratory samples (blood count, albumin, liver tests, CRP fecal calprotectin from stool) were taken. Each patient also underwent an endoscopy before study enrolment and was weighed. CDAI in patients with CD or MSS in patients with UC was counted.

The observed parameters (CDAI in patients with CD, MSS in patients with UC, CRP and other laboratory tests, weight and endoscopic findings) were assessed after 12 weeks of therapy.

In patients with CD, a partial response was defined as a ≥70-point decrease from the baseline in CDAI score and remission was defined as a CDAI score of <150. In patients with UC, a partial response was defined as a ≥2-point decrease from the baseline in a partial Mayo score and remission was defined as a total score on the partial Mayo index of ≤2 points. Patients who did not show a partial response or remission were considered non-responders.

The study protocol was approved by the ethics committee of the Second Faculty of Medicine, Charles University in Prague, Czech Republic and each patient provided signed consent to this study prior to being accepted.

### Statistical analyses

The variables were tested for normality by a Shapiro-Wilcoxon normality test and the differences between studied groups were analysed by either a Paired T-test or a Wilcoxon matched-pairs signed rank test. Fisher’s exact test was used to determine the association between two categorical variables. Statistical analyses were performed using GraphPad Prism version 6.0 (GraphPad Software, GraphPad Software, San Diego, CA, USA) and differences were considered statistically significant at p <0.05.

## Results

### Patient demographics (Table 1)

**Table 1:** Baseline patient demographics and clinical characteristics.

**Sex**	Men, number [%]	31 [62.0]
	Women, number [%]	19 [38.0]
**IBD**	Crohn´s disease, number [%]	34 [68.0]
	Ulcerative colitis, number [%]	16 [32.0]
**Age before therapy, median [range]**		39,5 [20.0–74.0]
**Age at diagnosis, median [range]**		27.0 [10.0–66.0]
**Site of disease in CD patients**	Colon only, number [%]	4 [11,8]
	Small intestine only, number [%]	11 [32.3]
	Small intestine and colon, number [%]	19 [55.9]
	Fistulas, number [%]	16 [47.1]
**Extent of disease in UC patients**	E1 (proctitis), number [%]	0 [0.0]
	E2 (left-sided colitis), number [%]	12 [75.0]
	E3 (pancolitis), number [%]	4 [25.0]
**Extraintestinal manifestations, number [%]**		15 [30.0]
**History of surgery, number [%]**		18 [36.0]
**Previous therapy**	5-aminosalicylates, number [%]	46 [92.0]
	oral corticosteroids, number [%]	50 [100.0]
	azathioprine, number [%]	48 [96.0]
	methotrexate, number [%]	8 [16.0]
**Concomitant therapy**	5-aminosalicylates, number [%]	30 [60.0]
	low dose oral corticosteroids, number [%]	16 [32.0]
	azathioprine, number [%]	24 [48.0]
	methotrexate, number [%]	1 [2.0]

CD, Crohn’s disease; CDAI, Crohn’s disease activity index; CRP, C-reactive protein; UC, ulcerative colitis.

In our study, fifty eligible patients (31 men and 19 women) with CD (34 patients) or UC (16 patients) were enrolled. The median patient age was 39.5 and the range was between 20–74 years of age. The median age of patients at first diagnosis and first manifestation of IBD were both 27.0 years.

In the CD group extent of disease was: only colon (4 patients), only small intestine (11 patients), both small bowel and colon (19 patients). Fistulas in CD patient´s anamneses were also reported: perianal fistulas (13 patients), enteroenteral fistulas (2 patients) and rectovaginal fistula (1 patient). In the UC group extent of disease was: E3 (pancolitis) was present in 12 patients and E2 (left-sided colitis) was present in 4 patients. Extraintestinal manifestations of IBD were reported by 15 patients (joints in 12 and skin in 3 patients). Finally, history of surgery was reported in 18 patients (ileocecal resection in 10 patients, right-sided hemicolectomy in 5 patients, stricturoplasty in 2 patients and subtotal colectomy in 1 patient).

Prior to biological therapy with biosimilar adalimumab, 46 patients had received 5-aminosalicylates, 50 oral corticosteroids, 48 azathioprine and 8 methotrexate. In terms of concomitant therapy during biosimilar adalimumab therapy, 30 patients were treated with 5-aminosalicylates, 16 with low-dose systemic corticosteroids, 24 with azathioprine and 1 with methotrexate.

All patients received either FKB327 (Hulio^®^, 22 patients) or GP2017 (Hyrimoz^®^, 28 patients) subcutaneously at Week 0 (160mg), Week 2 (80mg), Week 4 (40mg), Week 6 (40mg), Week 8 (40mg), Week 10 (40mg) and Week 12 (40mg) in a classical scheme.

### Crohn´s disease—Effectiveness

In the CD group (34 patients): 25 patients (73.5%) achieved remission and 4 patients (11.8%) partial response after 12 weeks of therapy. Four patients (11.8%) were classified as non-responders to therapy with biosimilar adalimumab. One patient from this group discontinued therapy prior to Week 12 because of an adverse event (paraesthesia of all extremities, based on EMG neurogenic lesion).

**Fig 1:**
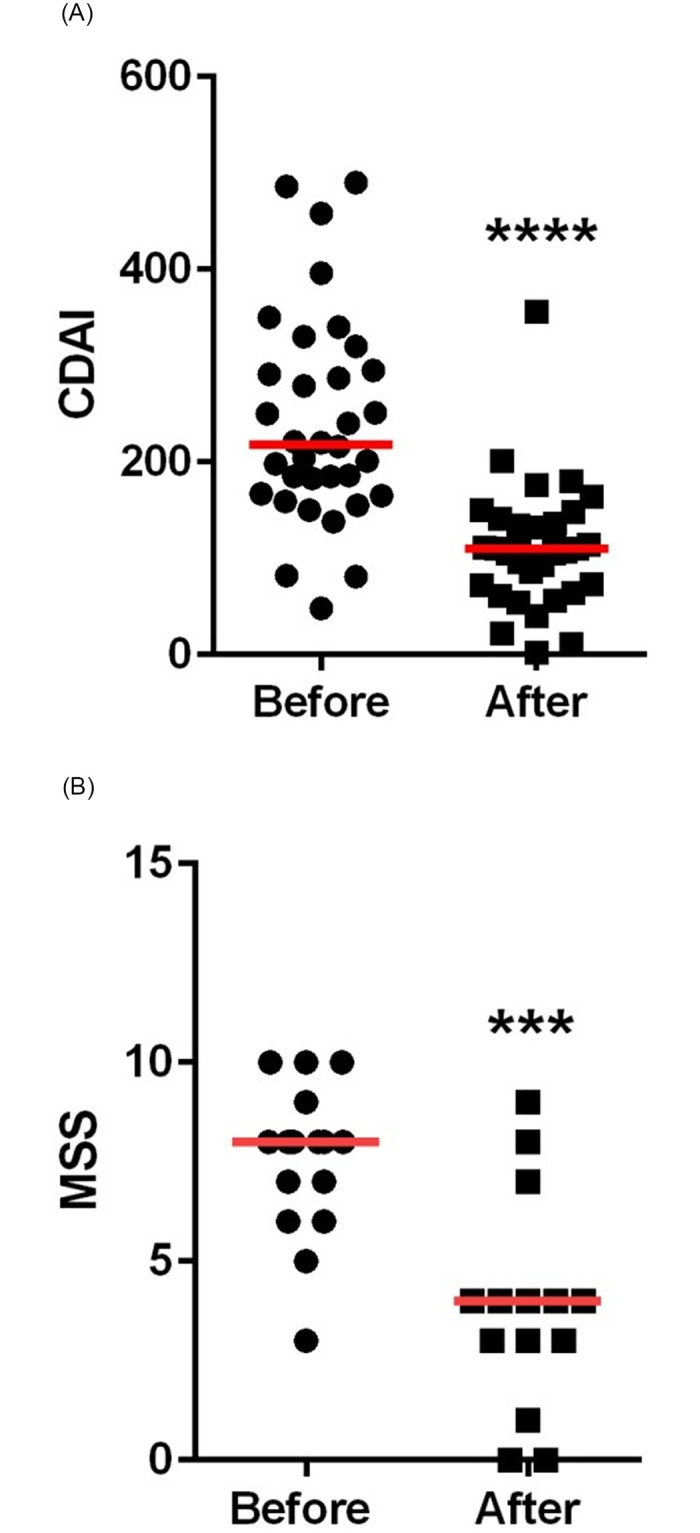
**A**: The trend of CDAI values in the CD group before and after 12 weeks of therapy. **B**: The trend of MSS values in the UC group before and after 12 weeks of therapy.

The median CDAI value in the CD group before therapy was 216.0 and this decreased to 110.0 (p<0.0001) after 12 weeks of therapy (Fig 1A).

### Ulcerative colitis—Effectiveness

In the UC group (16 patients), only 3 patients (18.8%) achieved remission and 7 patients (43.8%) partial response after 12 weeks of treatment. Four patients (25.0%) showed no response to therapy. Two patients discontinued therapy prior to Week 12, both due to allergic reaction.

The median MSS value in the UC group before therapy was 8.0 and this decreased to 4.0 (p = 0.0003) after 12 weeks of therapy (Fig 1B).

### Crohn´s disease and ulcerative colitis—Other parameters

The median of weight before therapy was 80.0 kg, but this decreased to 78.0 kg after 12 weeks of therapy.

**Fig 2:**
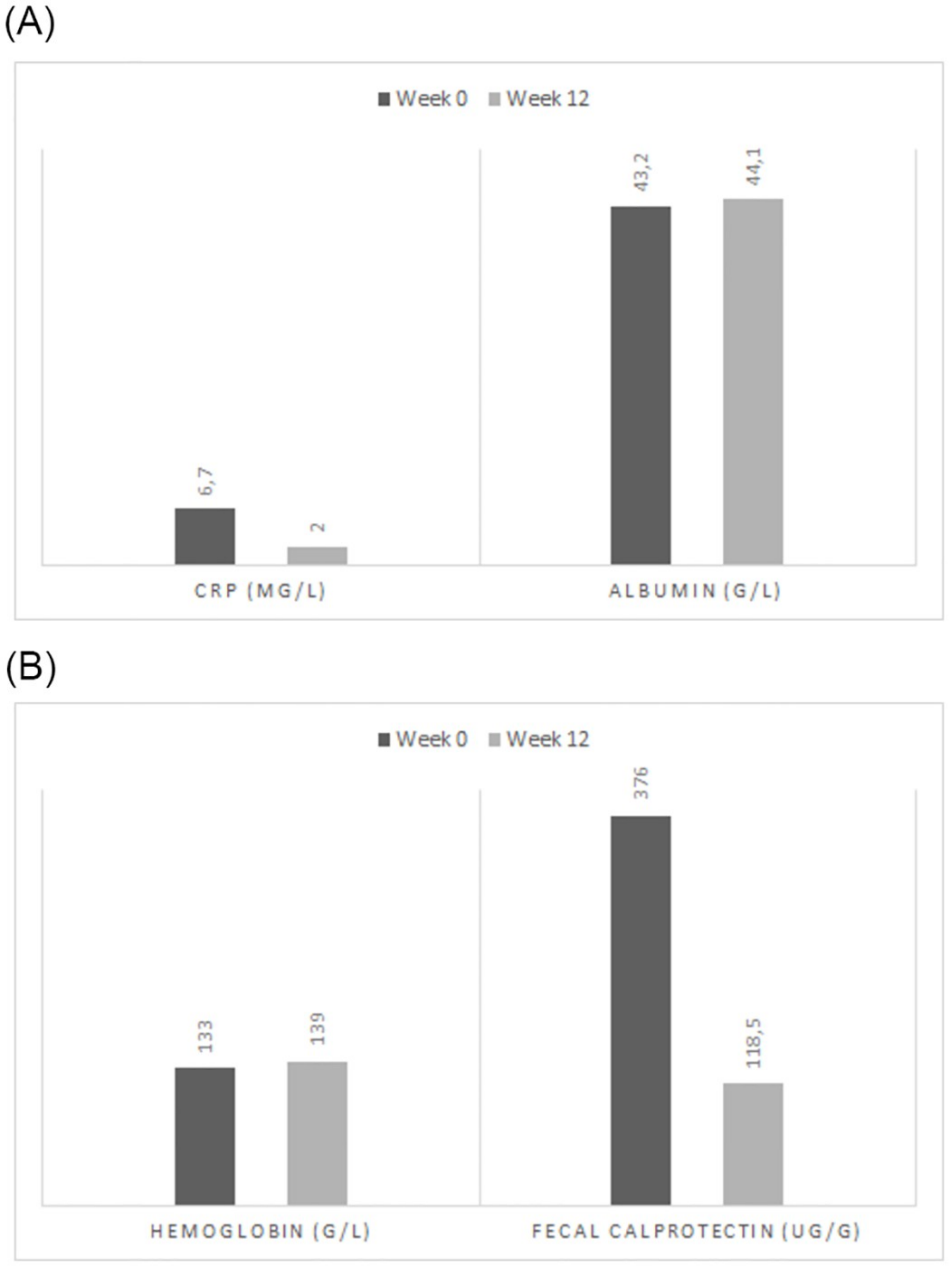
**A**: The trend of CRP and ALBUMIN before and after 12 weeks of therapy. **B**: The trend of HEMOGLOBIN and FECAL CALPROTECTIN before and after 12 weeks of therapy.

Trends in laboratory parameters were also compared before and in Week 12 of therapy: CRP 6.7 mg/l and 2.0 mg/l respectively (p<0.0001) (Fig 2A), albumin 43.2 g/l and 44.1 g/l respectively (p = 0.003) (Fig 2A), ALP 1.2 μkat/l and 1.1 μkat/l respectively (p = 0.8390), AST 0.4 μkat/l and 0,42 μkat/l respectively (p = 0.6260), ALT 0.4 μkat/l and 0,5 μkat/l respectively (p = 0.4543), GGT 0.6 μkat/l and 0.6 μkat/l respectively (p = 0.5794), total bilirubin 10.7 μmol/l and 9.0 μmol/l respectively (p = 0.7902), urea 4.2 μmol/l and 4.0 μmol/l respectively (p = 0.4127), creatinin 78.5 μmol/l and 80,9 μmol/l respectively (p = 0.5802), leukocytes 8.9 x10^9^/l and 8,2 x10^9^/l respectively (p = 0.0274), haemoglobin 133.0 g/l and 139.0 g/l respectively (p<0.0001) (Fig 2B), thrombocytes 330.0 x10^9^/l and 282.0 x10^9^/l respectively (p<0.0001) and fecal calprotectin 376.0 μg/g and 118.5 μg/g respectively (p = 0.0004) (Fig 2B).

In patients who achieved remission, the most noticeable effect was observed after the 6^th^ dose while in patients with partial responses after the 4^th^ dose of therapy.

### FKB327 (Hulio^®^) and GP2017 (Hyrimoz^®^) differences

In 22 IBD patients treated with FKB27(Hulio^®^) and 28 patients treated with GP2017 (Hyrimoz^®^), remission or partial response was achieved in 18 patients (81.8%) and in 21 patients (75.0%), respectively, after 12 weeks of therapy (p = 0.7344).

In the FKB27(Hulio^®^) group, the median of CDAI value in the CD group before therapy was 201.0 while the median of MSS value in the UC group before therapy was 6.0, after 12 weeks of therapy this decreased to 110.0 (p<0.0001) and 3.5 (p = 0.1250), respectively.

In the GP2017 (Hyrimoz^®^) group, the median of CDAI value in the CD group before therapy was 251.0 while the median of MSS value in the UC group before therapy was 8.0, after 12 weeks of therapy this decreased to 110.5 (p = 0.003) and 4.0 (p = 0.0056) respectively.

### Safety and tolerability

Seven adverse events were identified during the therapy: in four patients mild complications were observed (one patient with skin eczema, one patient with mild course of COVID-19, one patient with mild course of mycotic infection and one patient with mild course of pneumonia) and in three patients (1 with CD and 2 with UC) the therapy was discontinued as described above.

## Discussion

Adalimumab is indicated in IBD patients with: moderate to severe active CD that is refractory to conventional treatment, corticodependence and some extraintestinal manifestations; additionally, in CD patients with perianal fistulas and in UC as a rescue therapy in case of acute severe UC attack [19, 20]. The effectiveness of adalimumab RMP in induction of remission in patients with IBD has been demonstrated in many studies. The CLASSIC I study played a crucial role in demonstrating efficacy in inducing remission in patients with CD—a response rate at Week 4 was based on dosages of 40 mg/20 mg, 80 mg/40 mg, and 160 mg/80 mg 18%, 24% and 36% respectively [21]. The study ULTRA 1 in patients with UC played a similarly crucial role in the induction of remission—at week 8 18.5% of patients in the adalimumab 160 / 80mg group and 10.0% in the adalimumab 80 / 40mg group were in remission compared with 9.2% in the placebo group [6]. Aware of the differences between our methodology and these studies, our data showed in CD and UC high effectiveness of biosimilar adalimumab in inducing remission—in the CD group 85.3% of patients and in the UC group 62.6% of patients achieved remission or partial response. Our data suggest a poorer efficacy of biosimilar adalimumab in patients with ulcerative colitis.

The first biosimilar in IBD therapy was the biosimilar equivalent of infliximab (CT-P13), developed by Celltrion. CT-P13 was approved by the European Medicine Agency (EMA) in 2013. Many authors have provided data on the efficacy and safety of the biosimilar infliximab CT-P13 in patients with Crohn’s disease and ulcerative colitis [22–24]. Keil et al. provides evidence of the effectiveness of CT-P13 in IBD patients in induction of remission [23] and Schmitz et al. also proved efficiency and safety in 12-month follow-up [24]. The first biosimilar analog of adalimumab was approved by EMA in all indications of the original product in April 2017. Efficacy and safety data for biosimilar adalimumab FKB327 (Hulio^®^) and GP2017 (Hyrimoz^®^) for patients with Crohn’s disease or ulcerative colitis were extrapolated from efficiency and safety data in patients with rheumatological or dermatological problems [16, 17]. The available data on the efficacy and safety of the biosimilar adalimumab directly in patients with inflammatory bowel disease are limited and include, in particular, retrospective studies with a small number of patients. Kamat et al. (India) proved safety and efficiency in inducing and maintaining remission in 70 patients with ulcerative colitis or Crohn’s disease [25]. Efficiency in inducing remission was achieved in 46.9% of CD patients and 52.4% of UC patients and maintained remission over 1 year was achieved in 32.7% of CD and 33.3% of UC patients.

In our UC group of patients 18.8% of patients achieved remission and 43.8% of patients achieved partial response after 12 weeks of therapy. The data are consistent with the conclusions of Chandra et al. (India), who evaluated the efficacy and safety of the adalimumab biosimilar ZRC-3197 (Exemptia) in 25 patients with steroid-refractory ulcerative colitis. In this study clinical remission was achieved in 16% of patients at 12 and 24 weeks and clinical response in 48% of patients at week 12 and 44% at week 24 [26]. Chandra et al. also described a statistically significant decrease of MSS (MSS at baseline 10.16 points, MSS at week 12 5.72 points and MSS at week 24 5.52 points). These data are consistent with our results—the median of MSS value in our UC group before therapy was 8.0 and then decreased to 4.0 after 12 weeks of therapy with biosimilar adalimumab.

In our CD group 73.5% of patients achieved remission and 11.8% of patients achieved partial response after 12 weeks of therapy. Ribaldone et al. achieved more or less similar results in a study of 25 patients with CD naive patients to biosimilar adalimumab ABP 501—clinical response at 3 months was achieved in 60% of CD patients and clinical remission at 3 months in 56% of CD patients [27].

In line with our results, a comparison with the results of our previously performed study with the biosimilar infliximab CT-P13 and its effectiveness in inducing remission appears to be highly interesting [23]. The advantage is the possibility of a good comparison of both studies with respect to the similar design of these two studies. In a study with the biosimilar infliximab CT-P13 in patients with CD at week 14 remission was achieved in 50.0% of cases with partial response in the remaining 50.0%. In our recent biosimilar adalimumab CD group 73.5% of patients achieved remission and 11.8% of patients achieved partial response after 12 weeks of therapy. In the UC group, patients treated with biosimilar infliximab achieved remission in 40.9% of cases and partial response in 54.5%. In our recent biosimilar adalimumab UC group only 18.8% of patients achieved remission and 43.8% of patients achieved partial response. Based on these results, induction therapy with biosimilar infliximab CT-P13 appears to be more effective in inducing remission in patients with both CD and UC rather than induction therapy with biosimilar adalimumab. To the best of our knowledge, this seems to be the first comparison of biosimilar infliximab and biosimilar adalimumab in induction remission in biologically naïve patients with inflammatory bowel disease.

In our study, two types of biosimilar adalimumab were used: FKB327 (Hulio^®^) and GP2017 (Hyrimoz^®^). There was no statistically significant difference in the efficacy of induction therapy between these two medicaments (remission or partial response of 81.8% in FKB27 vs. 75.0% in GP2017). Only in the FKB27 group, the median of MSS value decrease was not statistically significant compared to other results—however, we do not consider this result to be clinically significant.

Many parameters were carefully monitored during our study. The evaluation of efficiency in our study was based mainly on the trend of activity indexes. Statistically significant decreases in values of CDAI in CD and MSS in UC were demonstrated in our groups of patients. In the CD group, the median CDAI before therapy was 216.0 and after 12 weeks of therapy decreased to 110.0. CDAI is one of the widely used clinical activity indexes, whose invaluable role has been demonstrated in a number of studies [28–30]. Also, within the UC group, the median of MSS value decreased from 8.0 to 4.0. MSS is relatively simple to use in clinical practice and also in clinical trials [31]. In addition to the response data collected in the current study, our patients also showed a significant decrease in CRP, fecal calprotectin and leukocytes values. Furthermore, increases in the serum albumin and haemoglobin were statistically significantly present. The elevation of CRP could be associated with higher clinical severity of disease much more strongly in patients with CD than UC—low levels of CRP do not rule out the presence of active disease, especially in patients with UC [32]. In patients with CD, higher levels may be associated with a higher risk of relapse or failure of biological therapy [33, 34]. Fecal calprotectin appears to be a very suitable marker for the evaluation of clinical and endoscopic activity of both CD and UC [35–37]. Zittan et al. clearly demonstrated the correlation between MSS and fecal calprotectin values [37], which agrees with our conclusions. Other monitored parameters (leucocytosis, haemoglobin and albumin) are commonly used markers of disease activity in a broader sense. These parameters do not have an irreplaceable role in patient monitoring and provided only additional information about the patient’s condition [38].

A biological therapy with antiTNFα therapy is relatively safe, but can be associated with potentially serious adverse effects. Seven adverse events occurred during our study—in percentage terms, 14% of patients in our group. Four of these were classified as a mild infection, which did not lead to discontinuation of therapy. Different kinds of viral, bacterial or fungal infections could be associated with the use of biological therapy with antiTNFα in IBD and can occur in up to one third of patients with antiTNFα [39, 40]. Therapy of biosimilar adalimumab had to be discontinued (two allergic reactions, one neurological complication) in three of seven adverse reactions (6% of all patients), which is practically the same as in the original adalimumab studies. In the CHARM study 6.3% of patients with CD discontinued therapy with adalimumab because of an adverse event [41]. In comparison to the study by Kamat et al. with biosimilar adalimumab, where the adverse reaction was present in 28.6% of patients and severe in 10% of patients, there were significantly fewer treatment-associated adverse reactions in our study [25]. On the other hand, in our study with the biosimilar infliximab CT-P13, adverse reactions were present in only 7.7% of patients [23].

Limitations of this study are mainly: a relatively small number of included patients, non-randomised and non-blinded design of our study. However, despite these limitations, our results show positive clinical outcomes following administration of biosimilar adalimumab to IBD patients.

In conclusion, our prospective observational multicentric study proved the effectiveness and safety of adalimumab biosimilars FKB327 and GP2017 in the therapy of patients with Crohn’s disease and ulcerative colitis. However, larger, double-blind, randomised, prospective, long-term studies are necessary to be done to provide further data about efficiency and safety of therapy with biosimilar adalimumab.

## Supporting information

S1 Table Source data of this study divided into three tables. (XLSX)
